# The Effects of the COVID-19 Pandemic on Educational Communities: Evidence of Early Childhood Education Students

**DOI:** 10.3390/ijerph19084707

**Published:** 2022-04-13

**Authors:** Miguel Martín-Sánchez, Jorge Cáceres-Muñoz, Cruz Flores-Rodríguez

**Affiliations:** Education Sciences Department, Teacher Training College, University of Extremadura, 10003 Cáceres, Spain; jorgecm@unex.es (J.C.-M.); cruzflores@unex.es (C.F.-R.)

**Keywords:** school, teachers, educational community, COVID-19, perception, health education

## Abstract

In the last two years, the effects of the COVID-19 pandemic on schools and its consequences for the training of new teachers have been the subject of numerous studies. The pandemic has led to a change in schools and their functioning, as trainee teachers have had to be introduced to a new environment for which university training proved to be insufficient. The pandemic poses shared challenges in which future teachers must be present. The objective of this study is to assess the perception of students enrolled in Early Childhood Education courses at the University of Extremadura (Spain) regarding the impact relationships between the subjects and agents of the educational communities. In order to achieve this goal, we present a qualitative study with a phenomenological design. The results of this research show perceptions in line with what the scientific literature shows: a profound change in the relationships between the different agents. Communication increased, but pedagogical issues were displaced by concerns about the health emergency. This study concludes with the need to broaden the knowledge of future teachers about the administrative functioning of and their relationship with the school, as well as about new resources to address new challenges.

## 1. Introduction

The health crisis resulting from the spread of COVID-19 transformed numerous social and individual spheres and is forcing institutions and citizens to adapt to a new reality. Many of these changes have offered an opportunity to introduce new dynamics and overcome old contradictions. This crisis has been an opportunity to reinforce global dialogues, as the best example of recent international understanding. It is introducing a new era of industrial relations, as well as in the context of work itself, causing workers to change their labor and life perspectives [1]. On the other hand, the crisis has introduced new problems, while reviving old ones. It is not only the apparent technological incapacity demonstrated by various institutions, unable to respond to citizens’ demands during the confinement, but also the material shortages that have arisen. In fact, COVID-19 has also led to instability and economic uncertainty, represented in markets, consumption patterns and available resources for development in multiple contexts and institutions [2,3].

One of the institutions that has suffered the most during this crisis is the school, which faces a double challenge: to continue offering quality education, while adapting education (contents and methods) to the new circumstances. The confinement introduced as a result of the COVID-19 pandemic was a critical moment for all education systems in the world that can be studied from multiple perspectives and from the approaches of many individuals. In this article, we aim to assess the perception that the students enrolled in Early Childhood Education courses at the University of Extremadura have of the impact that COVID-19 has had on the relationships between subjects and agents of the Early Childhood Education Educational Communities.

The results show that the perceptions of the respondents are very similar to what the scientific literature explains: the weakening of relationships between students, the fragility of family–school relationships, the accelerated and uneven digitization or overexertion of all active members of the educational communities. There are small variances on certain issues due to the diversity of socio-economic backgrounds. These similarities and differences are expanded upon and discussed in this paper. It is also noteworthy what the results show about the received university education: more than half of those surveyed stated that they did not feel prepared to face the educational challenges that COVID-19 poses for teachers.

The haste of the changes, as well as their impact and duration, has led to a gap between the demands and needs of students and the school’s capacity to respond to them. This has generated great stress on the educational community, as well as changes in the school and its methods, representing a new era, although not as profound as some thought [4]. Its effects are well known and have been documented over the last two years [5,6,7]. One of the most significant problems is the digital divide that makes it difficult for students with fewer material resources to face the emergency remote education process [8] that is introduced every time there is a new confinement [9]. It is also important to highlight the growth of mental health problems due to the intense psychological pressures and high uncertainty that students experienced as a result of the dramatic changes caused by the imposed restrictive measures [10,11], as well as the difficulties in language acquisition at an early age due to the use of masks [12].

In this context, schools face a double challenge. Firstly, they have to guarantee a quality and equitable education for all students, because a quality education involves guaranteeing a participatory school. Human beings develop in a state of community, and the school and the educational community are the result of human thought, which cannot be created if it is not one with others, of others among others [13]. Secondly, a quality and equitable school is a matter that affects students, as a democratic and participatory entity, which must rebuild social relations and participation, overcoming the limitations imposed by the pandemic. For the first challenge, the school must be aware of the shortcomings it already has, which partially emerge after moments of great tension, such as the quarantine experienced in 2020. The students’ families had to assume, to the extent of their possibilities, these shortcomings in material and human resources. In this way, the school fails in its institutional aspirations of quality and equity for everyone, leaving the education of citizenship to the material and cultural possibilities of the students’ closer environment.

For the second challenge, the school has to deal with a situation that already existed before the pandemic. As a socializing agent, the school has to introduce codes and customs to the new pupils. When codes emerge so quickly, customs must coexist with them, prompting a moment of dialectic contradiction for which the school must be very clear about its mediating role. The school must be clear about its position in the face of these challenges, bearing in mind the importance of participation in social contexts, which lays the foundations for the equality of opportunity, learning, and participation [14]. The relationships established between the different school subjects are being altered, causing a physical separation that translates into greater social distancing. This can be negative in a scenario of active social reproduction, where the fundamental knowledge of the society into which the pupils are being introduced will be built. Precisely because of this reason, a school based on the principles of participatory and active pedagogy offers an inalienable condition in the process of building an active and participatory future generation, one that is more egalitarian, supportive, and committed [15].

Finally, the pandemic has affected not only internal school relations, but also the relations that the school establishes with families and other educational and social institutions. In turn, the school, as an institution, must relate to other actors at the same level. These relationships have also been altered by the course of the pandemic, which has affected the implementation of various educational policies. The gap between the demands of the schools and the response capacity of the different institutions is widening.

### 1.1. COVID-19 in Spain and Extremadura

Since spring 2020, there has been a progressive reopening of schools in European countries, and in the case of Spain, the reopening of face-to-face education in schools took place in September 2020, applying the measures approved by the Interterritorial Council of the National Health System and the Sectoral Conference on Education on the Declaration of Coordinated Public Health Actions against COVID-19 for educational centers during the 2020–2021 academic year on 27 August 2020 and the document on Prevention, Hygiene and Health Promotion Measures on 17 September 2020 [16].

It should be noted, as a significant fact, that the trainees in Extremadura encountered a changing and volatile scenario. From the start of the academic year in September 2020 until December 2020, according to official data [17], hundreds of students were infected (more than 2500), teachers were on sick leave due to infection (almost five hundred, according to official data), and there were several closures and confinements of classrooms, which produced doubts and uncertainties. This period of new normality produced uncertainty in the face of the constant risk of classroom closures due to contagion. During the period in which these trainees were in the schools, the Autonomous Community of Extremadura implemented a strict pandemic control protocol, such as the complete confinement of a classroom when 3 or more positive cases were detected, or 20 percent of the class in groups with few students. This meant that, during that period (September–December 2020), the Junta de Extremadura decreed the closure of more than a hundred classrooms due to cases of coronavirus and the quarantine of dozens of groups [17], figures that changed and varied rapidly, inter- and intra-day, as there were constantly high and low levels of contagion. This situation is very significant, as the trainees had to face a very complicated situation in the classroom, especially when Spain was in the midst of the third wave of contagion, which produced an accumulated incidence per 100,000 inhabitants, on 8 November, of 601 in Spain and 630 in Extremadura [18]. These high figures had a significant impact on Spanish society in general and on the school population in particular.

In Spain, according to data from the Red Nacional de Vigilancia Epidemiológica (National Epidemiological Surveillance Network) [16], from 22 June 2020 to 1 June 2021, 3.2% of the total number of confirmed cases corresponded to children under 5 years of age, 4.3% to 5–9 years of age, and 11.9% to 10–19 years of age. Nationwide seroprevalence surveys were conducted to estimate the population affected by COVID-19 and found an overall prevalence of 9.9%. This would be the percentage of people in the population with IgG antibodies to SARS-CoV-2 based on the results of the sampling conducted between 16 November and 29 November 2020. In relation to the pediatric population, the prevalence given was higher than those obtained from cases detected, as they also included mild or asymptomatic cases. In children and adolescents, the following prevalence was estimated, according to age group: 0–4 years, 5.1%; 5–9 years, 7.4%; 10–14 years, 8.6%; and 15–19 years, 8.5% [16].

Outbreaks in schools had two major peaks in November 2020 and January 2021, coinciding with the peaks of the second and third waves of the pandemic in Spain. The number of outbreaks decreased during the Christmas period and then increased in the first weeks of 2021. Most outbreaks were detected in secondary education (36.1%) and primary education (25%) environments, and were generally small outbreaks with an average of six cases per outbreak [16]. These data justify the health and educational interest during a critical and exceptional moment in Spain’s school history that should also be analyzed from the educational experience of students and the future teachers who will work in post-pandemic contexts.

### 1.2. Impact of COVID-19 on Institutions

As we already pointed out, the quarantine caused by COVID-19 in March 2020 has had a significant impact on state institutions, which have been overwhelmed in their attempt to respond to the needs and demands that have arisen. The most visible effect has been on public health systems, unable to meet the peaks in demand during the crests of the various waves. Likewise, communication between citizens and the public administration was made even more difficult, due to telecare and the haste with which labor was carried out during this period.

During this early period, public institutions failed in their attempt to manage citizens’ demands and claims, leading to an increase in mistrust and inter-group conflicts. Institutions have the function of providing a sense of security to citizens and offering models to establish group norms. When these roles are neglected, and citizens perceive that their needs are not met, distrust of institutions leads to less trust and less cooperation and less pro-social behavior. It represents a decrease in trust between strangers, cooperation and pro-social behavior, while prejudice, conflict, polarization, and extremism increase [19].

This mistrust, which stems from a lack of institutional response or an unsatisfactory response, erodes community links, leading to further individual isolation. In extreme situations, this mistrust extends beyond the institutional sphere and moves into areas such as politics and science, giving rise to all kinds of denialist and anti-vaccine positions. In more general situations, disillusionment with institutions, coupled with the global quarantine, had numerous effects on citizens, from emotional health to cognitive performance, as well as changes in behavior and social conduct [20]. As we have been pointing out, the deterioration of the social network occurs to the extent that the units that make it up deteriorate.

We must not forget that behind the various phenomena that have emerged as a result of COVID-19, and which may explain the growth of related mental health problems, there are material factors that were already present before the pandemic. The most vulnerable groups, who were even more dependent on institutions, have been abandoned in many dimensions, due to the loss of jobs and benefits, the lack of primary health care, or the closure of schools. In short, all this produces a generalized sense of uncertainty about the future. In this sense, improving the material conditions of individuals contributes to improving their state of health, which in turn generates a more cohesive and productive citizenship. Maulik, Thornicroft and Saxena propose that the most vulnerable groups should be taken into account in the development of labor, financial and legislative policies [21]. This strategy implies extending beyond interventions and moving into the field of prevention. It is precisely neoliberal policies that have produced a wider social gap in recent years since the 2008 crisis, aggravated in the times of COVID-19, which have led to a drop in income and an increase in economic, gender, racial, cultural and educational inequalities for the most vulnerable groups [22].

The most severe effects described above were most prominent during the first year, which was marked by quarantine, economic recession and a lack of vaccines. Many of these effects were largely overcome with the start of the vaccination campaigns at the end of 2020. Since then, the public health effects of COVID-19 have been diminishing, while mobility restrictions are being lifted and the economy is starting to show signs of recovery. This is all having a positive effect on communities, which are beginning to change their outlook for the future. Despite this, institutions have undergone a significant detente that continues to impact their relations with citizens, as well as the relations between people and social agents themselves. In the following, we delve into the details and characteristics of these altered relations in the school context, which includes not only institutional relations, but also relations between the agents and subjects that coexist in this scenario.

### 1.3. Consequences of COVID-19 on Educational Community Relations

The relationships established between the different educational agents have a fundamental impact on the success of the teaching and learning process. In that regard, we are not only referring to school agents (pupils, teaching staff and families), but also to other educational agents that orbit the school and whose presence is essential. In this second group, we include the educational administration, local institutions (town hall, health center, police, companies, etc.), social institutions (NGOs, cultural associations, foundations, etc.) and non-formal educational institutions (sports, academic, etc.). One of the realities highlighted by the COVID-19 pandemic is the fragility of the market and its impact on education. For decades, there has been a consumerist and mercantilist pedagogy based on individuality and mercantile competitiveness. This has turned the neoliberal ideology into doctrine, skewing relations within the educational community in terms of performance [23], leading to a serious crisis following the closure of all economic activity and its impact on family and school relations, which have not been able to keep up with traditional demands. Therefore, the way in which all these agents relate to each other and to schools, teachers, students and their families determines, to a large extent, the academic success or failure of students. This is why it is relevant to take a closer look at the state in which the pandemic has left inter- and extracurricular relations. As we have said, this is a factor that determines school success. It is an opportunity to learn more about the relational processes that take place in the school environment in order to improve them and take advantage of them to open up new ways of understanding. This will help us to achieve a deeper vision that will allow us to offer new perspectives to address the significance of school relationships, beyond the vicissitudes imposed by COVID-19.

As we have already mentioned, we focus on two sets of relationships: firstly, the relationships between students and their families, the school, and among students themselves. Secondly, we focus on the relationships established between the school and other institutions. The starting point is the beginning of the quarantine, when teaching became virtual and relations continued telematically. The first impact was on the students. The closure of schools to contain the spread of the pandemic led to an abrupt separation of the youngest students from their peer group. These early relationships are fundamental to the acquisition of social and communication skills, and in a quarantine context, the acquisition of cultural values and norms formulated by older generations becomes more difficult [24]. Moreover, the implementation of sanitary norms and, in particular, the use of masks are causing difficulties in the acquisition of language skills by pupils [8].

In addition, the quality of home schooling is determined by the amount of time families can devote to their children’s education, as well as their economic and cultural capital [25]. In general, a low socioeconomic status is associated with higher levels of anxiety and depression experienced by youth during the quarantine, while the cessation of school and extracurricular activity had no correlational impact [26]. Quarantines forced many families to live together for months at a time, which had a significant impact on parenting styles. Many families were forced to reformulate their own rules to adapt to the demands of the new context. The quarantine was also an opportunity to strengthen intra-family relationships.

In this relational kaleidoscope, teachers are the link between families and institutions, while also playing an active role in the teaching and learning processes. As a result of the pandemic, confinements and the return to the classroom with new safety measures, their work was shaken and implicated by numerous threats and constraints. Their presence is essential to the educational and socializing processes of the school environment. In this context, teachers can foster dialogue, mediate conflicts, offer support and guidance, act as an attitudinal reference and even provide psychological support [27], because teachers address and produce positive teaching–learning situations and build safe and attractive learning environments, overcoming students’ problems and social and school exclusion [28,29].

Furthermore, a smooth and positive relationship between teachers and families leads to better academic performance for students. This happened both during and after the quarantine [30]. The relationship among teachers themselves, which was eroded by the pandemic, is also important. Teachers had to deal with a return to schools marked by the challenges of the extra workload of adopting new safety measures, adapting lessons and coping with the digital environment, while contending with the increased anxiety they themselves experienced [31].

The pandemic increased three of the most implicated factors in the generation of stress in teachers: workload, emotional fatigue and educational reforms [32]. In that respect, although there has was not a total abandonment by the different administrations, the quarantine made access difficult. This leads to a lack of institutional attention that increases the feeling of abandonment of the teaching staff. As with other professionals, whose labor has been affected by the pandemic, the service they can offer to the public is gradually deteriorating, resulting in a government run by overstretched workers.

Finally, we set out to explore the perceptions that undergraduate Early Childhood Education trainees had of their own work, their training, and their abilities to meet the present challenge. Studies have identified feelings of insecurity, melancholy and uncertainty among undergraduate students in Primary Education courses, while also pointing out the consequences that training away from the school culture, which provides professional identity, can have [33]. This was also perceived by the teachers who took in the trainees during the quarantine period, who also warned of the problems associated with learning a profession far removed from it [34].

Presence, in this phase of training, means connecting with the closest reality that the future teacher has to face. Quarantine and virtual teaching produce a loss of meaning in the educational processes that they experience. This leads to feelings of frustration and lack of confidence in their own abilities, a circumstance that is even worse in the case of undergraduate teacher trainees in Physical Education courses [35]. The practicum, unlike ordinary classroom learning, is an opportunity to discover the reality of a profession that is changing due to the digitalization of schools and the introduction of new resources and methodologies. It is also important to know if they feel that their training enables them to face the new educational challenges of an individualistic society in order to transform it into an inclusive, participatory and equitable society, for which actions are needed that involve all educational agents to understand the new reality and enable positive changes in the community [36].

## 2. Materials and Methods

### 2.1. Study Design

This qualitative research [37,38,39] with a phenomenological [40] case study design [41] aims to assess the perception of Early Childhood Education undergraduate students at the University of Extremadura (Spain) on the impact that COVID-19 had on the relationships between subjects and agents in educational communities. A purposive sampling was used, whose selection criteria were: (a) being a student of the Degree in Early Childhood Education at the University of Extremadura; (b) being enrolled in the Health Education subject of the Degree’s syllabus; and (c) having taken the Practicum I subject of the syllabus in the 2020–2021 academic year.

The process of analysis focused on the perceptions of the population of students in initial training for their professional practice at the University of Extremadura (Spain). The participants in this study were 62 students who carried out their Practicum in different educational centers in the Autonomous Community of Extremadura (Spain) in a context marked by the pandemic, specifically 54 women (87%) and 8 men (13%) aged between 19 and 26 years (7.5%, 19 years; 45%, 20 years; 20%, 21 years; 2.5%, 22 years; 7.5%, 23 years; 2.5%, 24 years; 12.5%, 25 years; 2.5 %, 26 years), all of whom were enrolled in the Degree of Early Childhood Education at the University of Extremadura. The specific profile of the participants was a fundamental criterion in the design of this research: they were students who carried out their practical training on-site in schools during the major phase of adaptation and implementation of measures in teaching in the face of the virus. We are referring to the period between September and December 2020, the start of the school year experienced in Spain after the confinement, and the beginning of a new school era marked by life in the new normality.

**Figure 1:**
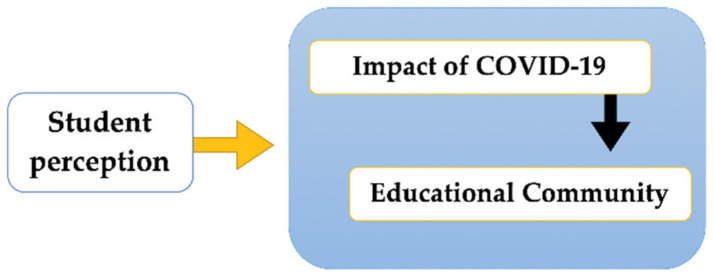
Research purpose. Self-made schemes.

The hermeneutic exercise that is practiced in this research allows, from the subjectivity of the actors present in the process, to find an understanding of the potential of the experiences of the protagonists [42], in this case, educational, social and health, to better understand the impact of COVID-19 on the health of the educational communities. Additionally, teleologically, the application of the qualitative methodology allows the testimonies of the practices to be interpreted for the purpose of knowing the reality better [43]. In order to understand this adequately, it is necessary to turn to the context and the cultural matrix of the one who produces the text (in its broad sense as a source), and to understand that hermeneutic rationality and certainty are achieved “by turning to the linguistic and communicative terrain, when we appeal to the texts themselves, to the words, to the arguments of rhetorical discourse, to the sense and to the intersubjective meanings—the conviction by the word” [44] (p. 61). In this case, we appeal to the knowledge of the perception of teachers in their initial training of the impact of COVID-19 on the relationships among members of educational communities (Figure 1).

### 2.2. Data Collection Procedure

In relation to the survey technique, the instrument used for data collection was a structured questionnaire [45] with open-ended and qualitative questions in order to offer a greater degree of freedom to the participants [46]. Questionnaires are an efficient data collection technique in educational research, as they are a technique with standardized procedures with which data are collected and analyzed from a sample in order to explore, describe and explain a series of characteristics [47].

The voluntary questionnaire consisted of 10 items of open questions, focused on different aspects of students’ perceptions of the relationships that the community experienced during the pandemic. It was validated by three experts in the field of education, who are professors at Spanish universities. We use the Google Forms application. Specifically, the questions focused on obtaining data along five main lines:

- Axis no. 1. To find out the undergraduate students’ perception of the impact of COVID-19 on the relations between actors and agents within educational centers.
- Axis no. 2. To find out the undergraduate students’ perception of the impact of COVID-19 on family cohesion and relations between families and schools.
- Axis no. 3. To find out the undergraduate students’ perception of the impact of COVID-19 on the relations among educational centers, the educational administration and community institutions.
- Axis no. 4. To find out the undergraduate students’ perception of the impact of COVID-19 on the relations among students, families and other educational or social institutions.
- Axis no. 5. To find out the undergraduate students’ self-perception of their level of training in relation to the impact of COVID-19 on subjects, agents and institutions in educational communities and their community environment.

**Figure 2:**
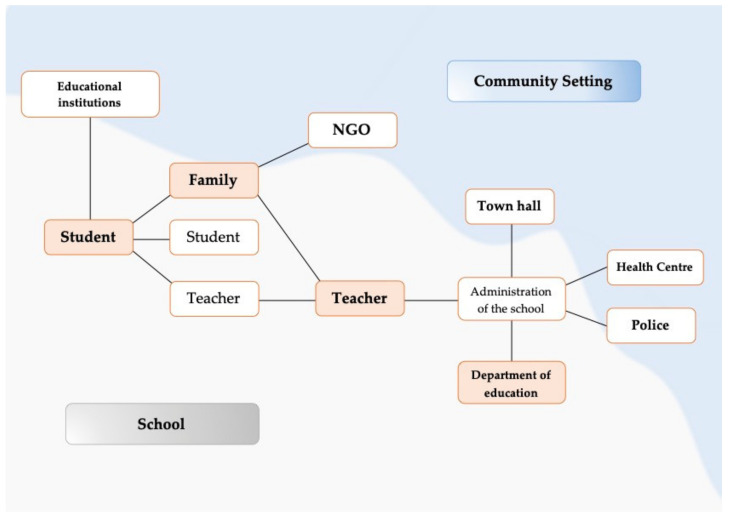
Educational community. Self-made schemes.

The questionnaire, based on this proposal, concerns the six main actors for which data are processed (Students, Teachers, Families, Educational Administration, Community Environment and Students in initial training), in order to know the students’ perception of the impact of COVID-19 on the educational communities. The configuration of the educational communities is based on the flow of communication, participation and interrelation among the different actors listed, all influenced by the health situation (Figure 2). The ten questions in the questionnaire were structured in the following sequence:

1. Academic relations among the students themselves (Axis no. 1).
2. Relationship between students and their teachers (Axis no. 1).
3. Relationship and organization among teachers, and between teachers and school governing bodies (Axis no. 1).
4. Educational relations between teachers and families (Axis no. 2).
5. Relationships, links and parenting styles between children and their families (Axis no. 2).
6. Relations between educational administration (Consejería de Educación, Inspección educativa) and educational centers (Axis no. 3).
7. Relationships between families and pupils with other complementary non-formal educational institutions (sports, artistic and academic) other than educational centers (Axis no. 4).
8. Relationship between families and social institutions (NGOs, associations, foundations, etc.) of the community (Axis no. 4).
9. Relations between local institutions (health centers, town hall, police, companies, etc.) and schools (Axis no. 4).
10. Level of training of trainee teachers in the context of the pandemic for professional practice in the educational community (Axis no. 5).

### 2.3. Ethical Procedures

There was an explicit consent that the students participating in the study voluntarily accepted for the dissemination of the data for scientific purposes. Informed consent was obtained from all subjects involved in the study. Confidentiality was maintained by responses being completely anonymous and only aggregated data are presented. Data for the current study were collected within an accepted educational setting and as part of the normal educational practices with opportunities for learning. Furthermore, the information obtained is encoded in such a way that, directly or indirectly, the identifiers for the subjects cannot be readily determined.

### 2.4. Quaitative Data Analysis

After collecting the participants’ responses, an analysis of the data was carried out, using the qualitative analysis software Atlas.ti 9. The use of software allows researchers to process large amounts of data in a systematic, reliable and efficient way [48]. With the use of this tool, a data dump was carried out, establishing a single document in which all the respondents’ testimonies were sorted for analysis. Next, a coding and categorization procedure typical of this type of methodology was carried out [49]. As Ruiz Olabuénaga points out [50], this requires a scientific reading, that is, a process of systematic, objective, replicable and valid reading of the students’ responses. Thus, the different answers given by the students about the actors in the educational community were analyzed deductively.

The tool used made it possible to carry out an analysis of the discourses and organization of the students’ testimonies, which led to a process of interrelation through the networks and concurrences inherent to the method. At the same time, a continuous process of methodological revision was carried out through Memos, which increased the coherence and stability of the data processing and the consistency of the analysis. The export of results—thanks to the software options—allowed for the orderly drafting available in the following section. The second phase of this research design involves a process of comparing the results with those published in the scientific literature. Thus, in the section on the discussion of the results, we present an accurate assessment of the levels of perception of undergraduate students in relation to the impact of COVID-19 on the community. These levels of perception are estimated on a scale of “high”, “medium”, or “low” levels of adjustment, depending on the similarities of the arguments put forward by the students and the reality contrasted by the scientific community. This will contribute to strengthening the methodological consistency of this study.

## 3. Results

### 3.1. Students’ Perception of the Impact of COVID-19 on the Relations between Actors and Agents within Educational Centres

**Figure 3:**
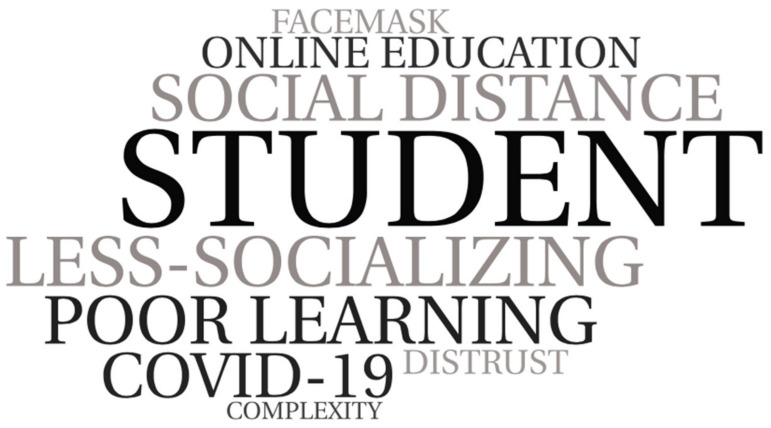
World cloud alluding to arguments in relation to students. Self-made schemes.

Student testimonies suggest that COVID-19 had a negative influence on the relationships among the students themselves (Figure 3). This perception is based on comments in which the respondents express that there has been a negative impact on the socialization of the student body, an increase in distrust and fear among them, and in some cases, episodes of anxiety and tension, which attenuated over time. The feeling of strangeness flooded the space and the forms of relationships, which became more complex. In addition, they argue that moments of cooperation and teamwork decreased substantially, so the possibilities for shared learning diminished. In addition, there are difficulties in oral expression. This perception is accompanied by an explanation about the causes, derived from the health situation. They point to the new organization of the center’s spaces and resources. The bubble groups, the obligatory use of masks, the increase in online learning experiences, the ban on the exchange of school material, the reduction in the number of pupils per classroom and less space for pupils to work and move around are the most prominent. Additionally, they warn of the previous situation, which was marked by a long period of confinement, with a high level of social distancing among pupils and which was also marked by a high prominence of screens as a substitute for the direct interaction among peers.

Furthermore, they say that the relationship between pupils and teachers also changed. However, this change does not coincide with a loss of affective bonds or trust between pupils and teachers, but with changes in the way in which this relationship is expressed. These relationship formats are subordinate to the biosecurity measures implemented in schools, which led to a greater distance, greater complexity and some communication problems. On the other hand, they argue that the range of flexibility in the educational relationship increased as a result of the use of online resources and (more individualized) attention to pupils due to the reduction in the number of pupils per classroom.

**Figure 4:**
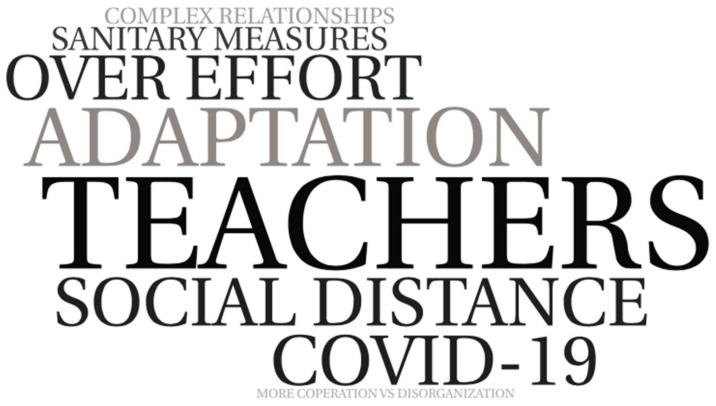
World cloud alluding to arguments in relation to teachers. Self-made schemes.

In the relation with teachers, the testimonies of the students show that they suffered the impact of COVID-19 on their relationships with the different actors in the school and the community (Figure 4). Excessive care in hygiene and compliance with measures were triggers for changes in the dynamics of day-to-day relationships. Along with this, there is a perceived overload in the efforts to adapt the lessons to virtual teaching moments and the individualized character that affect teaching styles, less close and to the detriment of cooperation.

Additionally, according to the undergraduate students, teachers, in their internal relations with their peers, also experienced different situations that affected the relationship and management dynamics of the schools. The arguments of the undergraduate students are divided into two clear positions. There are those who perceive that the health situation led to a forced distancing, mediated by self-protection measures and new technologies, resulting in a perception in which teachers are less organized, have difficulties in communicating fluently, feel less willing to reach an agreement and resort to improvisation. On the other hand, there is the opposite perception to the one just mentioned, in which the teaching staff and the management team strengthened their links, alternative cooperation measures were proposed to alleviate the initial lack of control, they developed support in extreme situations (absences of colleagues and improvement in methodologies) and self-training initiatives were produced to improve competences in the digital field:

“These relations have been very close, working as a team to achieve a common goal, to survive COVID and not to lose the educational part on the part of the teachers. In addition, we work quickly and efficiently by specifying all the agreements in a public document where both teachers and students can adapt their teaching and the general public can find out about developments and the new rules to be followed”.

Subject no. 14.

With regard to contact with families, undergraduate students report a deterioration in the relationship between them and teachers, as a relationship that is now distanced or cooled, due to the prevalence of online meetings, telephone calls and e-mails, which became constant substitutes for the traditional mentoring and face-to-face meetings. Additionally, the number of family members who can attend the few face-to-face meetings was limited to a minimum. This results in less close contact, only maintained in cases of family members of pupils with educational support needs. This is not a unanimous perception, but it is a majority one that is complemented by another perception: the relationships were closer during confinement. However, with the reopening of the schools, this relationship cooled.

### 3.2. Students’ Perception of the Impact of COVID-19 on Family Cohesion and Relations between Families and Schools

**Figure 5:**
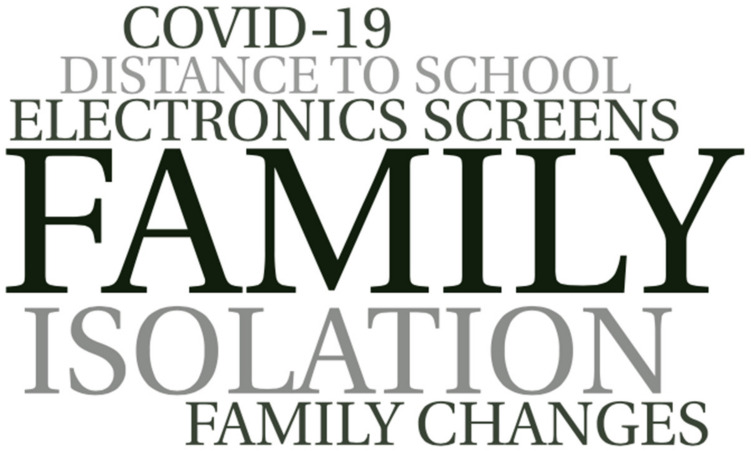
World cloud alluding to arguments in relation to family. Self-made schemes.

The students’ testimonies about the family connection reveal a very diverse universe (Figure 5). There are students who do not feel qualified to have a perception of the impact on the family institution, alluding to its complexity and heterogeneity. Those who do have a perception highlight a bipolarity marked by the bonding/deterioration of intra-family relations. Additionally, they perceive that family links were strengthened, and the time they spend together increased their value. However, they also perceive certain problems related to episodes of emotional instability, distancing—especially for fear of infecting older members of the family—and changes in the way parents care for their children, marked by the excessive use of electronic screens and teleworking.

Furthermore, as pointed out in the previous section, students have the perception that relations with educational institutions were impoverished. This can be perceived from two perspectives. Firstly, the climate of unease and tension in many families who feel isolated due to the lack of digital competencies to maintain the link with the new dynamics of online communication with the school. Secondly, the impossibility of carrying out activities or initiatives in which family members could come to the school and participate in their children’s learning processes and dynamics, as was the case in the past:

“The relationship between the school and the family is now less close, as parents cannot participate in school activities because of COVID-19 measures, nor go to talks, nor attend the school day, nor participate in projects. And now teachers and parents contact each other through e-mails or video calls, when previously meetings were held in person”.

Subject no. 26.

### 3.3. Students’ Perception of the Impact of COVID-19 on the School’s Relations with Educational Centres, the Educational Administration and Community Institutions

**Figure 6:**
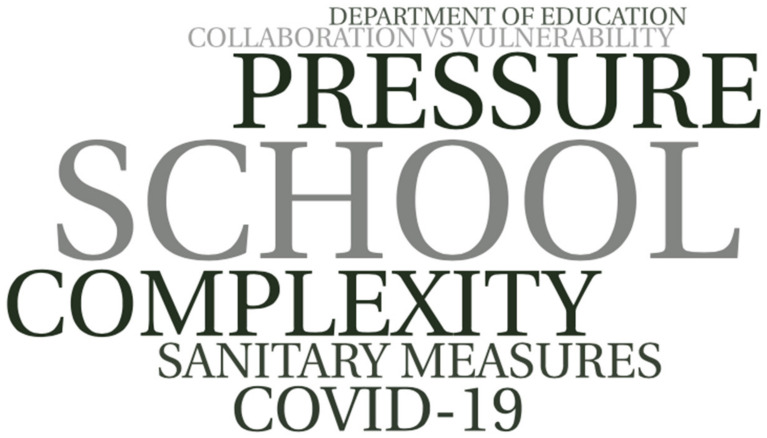
World cloud alluding to arguments in relation to school. Self-made schemes.

This sub-section aims to describe the undergraduate student’s perception of the relationship between the educational administration, personified in the Consejería de Educación (Regional Ministry of Education), educational inspectorates or any other agent managing school policy, and the school communities (Figure 6). In this sense, the testimonies given by the students put forward a common idea: the importance of support and close collaboration with the provincial or regional educational administration and the complexity and contingency that marks this relationship at the present time. From this point, testimonies allude to a lack of knowledge of this reality, but also to an explicit perception that is subdivided into three aspects: firstly, those who argue that the relationship did not changed; secondly, those who postulate that the relationship became closer in search of a common horizon within a framework of cordiality, with the presence of inspectors in the centers, cooperation and constant information about the safety measures to be implemented; and thirdly, those who argue that the relationship deteriorated and distanced themselves due to the prominence of online cooperation formats, the lack of consensus among the parties and the emergence of a certain feeling of helplessness in the educational centers:

“Personally, the public administration has had a relationship with regard to the capacity and the rules that had to be carried out in the school. It has also decided when it was necessary to close a school, in my case they closed it for several cases for 10 days. However, they often dictate the rules but if these rules generate a problem they do not give you a solution as such, so the school often has to investigate and look for a solution independently”.

Subject no. 5.

### 3.4. Students’ Perception of the Impact of COVID-19 on Relations among Students, Families and Other Educational or Social Institutions

**Figure 7:**
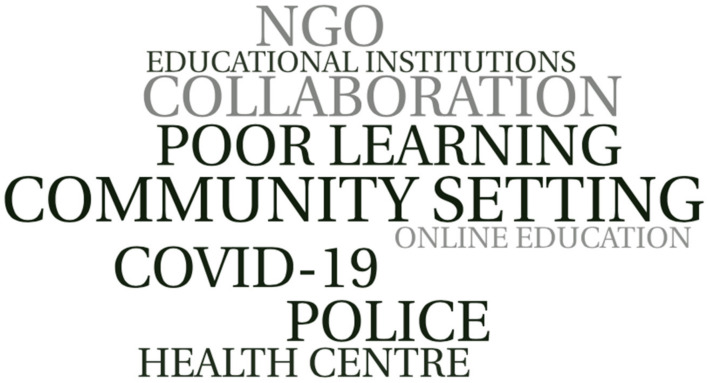
World cloud alluding to arguments in relation to the community setting. Self-made schemes.

The impact of COVID-19 on the community must also be understood in terms of the role played by non-formal educational alternatives that act in the everyday life of the community, social institutions and local public bodies. In relation to this, the perceptions that undergraduate students have in this regard show an awareness of the changes produced in these institutions (Figure 7). The students consider that the offerings of educational activities complementary to school was affected by the virus. Specifically, they identify a period during which their activity was non-existent, and then became moderate before reaching a current situation of stability in the context of a new normality. They identify, therefore, the impact it had on the community: The students consider that many parents were afraid to take their children to these activities, or that they decided to change to virtual training modalities. In addition, they warn of the loss of learning that this entailed for many children, who have not seen their training and personal development supported and complemented due to periods of restricted mobility.

With regard to the social institutions of the environment, the undergraduate students consider that these have been a social buffer for situations in which many families had needs. In general, this relationship was strengthened. However, they note that, in many cases, many families were the ones who helped certain social organizations to fulfill their task of support, resulting in a mutual help phenomenon:

“Many families have turned to NGOs and other institutions because for them it has been a case of all this, and the families that have been able to help have shown their support so that these institutions can continue. And also many NGOs have helped disadvantaged families”.

Subject no. 44.

Therefore, in general terms, they consider that this section of relations, far from being impoverished, was enriched.

Finally, as far as local entities are concerned, the students highlight the work of many city councils, security forces and law enforcement agencies. They emphasize that the relationship was maintained and that aid and collaboration were provided every time it was necessary, particularly in helping to maintain protocols related to virus detection and security in schools. Related to this, many emphasize that, despite the saturation of health centers, there has been collaboration and communication, which is a good example of the efforts made in times of contingency:

“These relationships have been very close, as in many schools the police control the crowds that tend to form at the entrance or exit of the school, the town hall also controls the entrance and exit of the school as in many cases they can cut off the street, the health centers also played an important role, as through them the schools could find out if there was a case of COVID in the classroom and how to deal with the situation”.

Subject no. 38.

### 3.5. Students’ Self-Perception of Their Level of Training in Relation to the Impact of COVID-19 on Subjects, Agents and Institutions in Educational Communities and Their Community Environment

This sub-section aims to describe the undergraduate student’s perception of their self-knowledge regarding their professional and social competence to deal with the reconstruction of the links of the school community in which they work. In this sense, more than 60% of the students perceive that they do not feel capable of dealing with the impact that COVID-19 produced in the relations of the educational community, compared to approximately 30% who do feel competent to do so and 10% who do not know how to position themselves in the face of this dilemma. In general, they feel that the experiences they had at the schools during their practicum experience period helped them to deeply understand the situation of collapse and overexertion experienced by the schools. Many consider themselves, thanks to this, more mature and with a greater capacity for attention and understanding of the variants of the current problems. In addition, they refer to the personal and professional growth that allowed them to have a greater number of skills for educational assistance.

“We were “fortunate” because my internship coincided with the COVID-19 pandemic and I was able to observe how the relationship between school communities and their environment has changed compared to before the pandemic. I have learned and gained a lot of experience that can help me in the future as a teacher. The educational aspect that I would reinforce would be good health education for children, which will serve them in the future and which should be instilled from an early age”.

Subject no. 7.

However, many agree on the problem of the durability of this situation, the unknown of how long the emergency health situation will last and how to adapt to the different stages of the virus. This opens the door to the following reasoning among many students: the need for continually updated training as well as further education on the virus.

**Figure 8:**
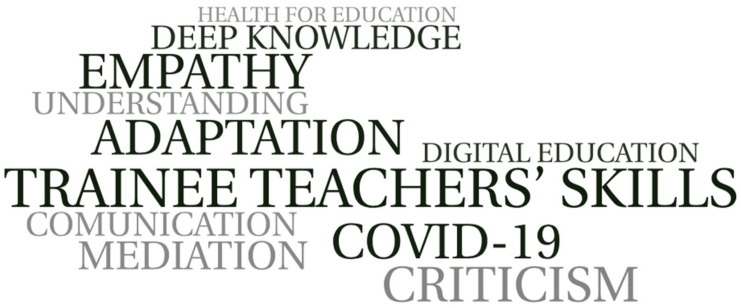
World cloud alluding to arguments in relation to trainee teachers’ skills. Self-made schemes.

Alongside this, students list a number of other areas to be strengthened in their training that they believe could help to improve their ability to revitalize the educational community. These areas are: health education and knowledge of childhood illnesses; improving the ways and means of communication among the actors interacting in the educational community, and among members of the community environment (associations, town hall and other local entities); increasing the range of knowledge in the field of digital education to prevent occurrences of isolation or under-communication; knowledge of strategies to improve coordination channels between professionals and the administration; reinforcing strategies for conflict resolution and dialogue between actors; and deepening the mastery of didactic strategies for programming and the use of materials and resources in extraordinary contexts, such as pandemics (Figure 8).

## 4. Discussion

There is no doubt that the COVID-19 pandemic, caused by the SARS-CoV-2 virus, changed the modus vivendi of millions of people around the world. We are in a situation of health, social and educational emergency. The quarantine in 2020 was a milestone in the history of education. Two years after the start of the COVID-19 pandemic, almost half of the world’s learners are still affected by partial or total school closures, and more than 100 million children will not reach the minimum reading level as a result of the health crisis [51]. The school, as all state institutions, has reconfigured its internal nature to serve its institutional function, which implies changes in its internal organization and management to reconfigure the way it relates to citizenship. Similarly, the institutional actors that relate to the school have also undergone changes, altering not only the way they relate to the educational institution, but also the type of synergies that are established between them. Thus, the changes that the pandemic has brought about, and which are still ongoing, are altering the nature of relationships in the school environment. A context that the new trainee teachers are entering with no other previous references than their time at the Infant School.

The introduction of these students to the school culture is a fundamental and significant process in the training of new teachers. At this stage, the codes of action, the system of values, and the meanings of the reality of which they are going to form part are configured. When this entire cultural universe is altered, we run the risk of introducing new teachers into an uncertain and changing context. However, it is necessary to stress the idea that, despite the difficulties and uncertainty of the health situation, education is opening up, becoming informalized [52], expanding and surviving.

Apart from that, the way in which higher institutions decreed school rules in the midst of the pandemic has projected a generalized sense of bewilderment that has only increased the uncertainty felt by school agents [30]. With these issues in mind, we are going to determine how close the perception of trainee teachers is to the reality of the scientific literature reviewed. The rapprochement will be determined by taking into account three levels of affinity: high, medium and low.

Firstly, trainee teachers were asked about the impact of COVID -19 on students. Their perception is quite close to what the scientific literature identifies. In particular, difficulties in the acquisition of social skills stand out, something already indicated in other studies [21]. The teachers identify an increase in feelings of mistrust, fear, anxiety and tension caused by the quarantine period, changes resulting from the new health regulations and the loss of emotional ties. They also detect difficulties in mastering certain communication skills due to the use of masks and social distancing. This corresponds with the results of the Charney, Camarata and Chern study, which warned of the problems that emanate from the use of the mask in the process of language acquisition [12]. A context that is even more aggravated in the case of pupils with mental and physical disability.

The second issue we looked into is the changes in the relationship between the teaching staff and the educational community. The undergraduate students in Early Childhood Education perceived an increase in the workload of teachers due to the numerous curricular and organizational adaptations they have faced. These changes were marked by social distancing and the individualization of teaching due to online teaching. This represents a double threat. Firstly, the absence of the teacher’s physical presence in socialization processes can lead to a lack of attitudinal references in students. Teachers also encourage situations of dialogue and peaceful resolutions, and likewise provide psychological support [24]. Secondly, the challenges encountered have led to tensions among the teaching staff, who sometimes are overwhelmed by the situation. In general, trainee teachers identified this situation, although others also noted the opposite: greater cohesion among the teaching staff in dealing with emerging needs, lack of staff, updating their knowledge and technological skills, and establishing new forms of organization. This disparity may be related to technological resources and school management. The less experience they have, the greater the difficulties in dealing with a new situation, with no previous experience to learn from, and with a saturated administration.

Regarding the relationship between teachers and families, respondents perceived an increase in the frequency with which they met, especially during the pandemic. However, these meetings tend not to be face to face, but via videoconference. The social and health situation occupies a large part of the meetings, with less time being devoted to educational issues. Serrano-Díaz, Aragón-Mendizábal and Mérida-Serrano also identified an increase in meetings between teachers and families, and found that good relationships between teachers and families lead to the better academic performance of students, even in the pandemic context [27]. This creates a gap between those who have sufficient resources and knowledge to maintain fluid communication and those who do not have sufficient resources [9,22]. The closure of schools during confinement, the educational and technological gap, and the lack of participation and relationships are some of the threats observed among participants in this study and other research related to educational participation in times of pandemics that demonstrate the need for communication and participation as guarantors of social and educational justice [53].

The third question, on the relations between schools and the education administration, was the result of a disparity of personal opinions. All agree on the need for closer ties so that the administration would have a better understanding of the reality of schools and so that the measures taken would be quicker and more efficient. Otherwise, respondents say that relations either did not change, or worsened due to distancing and lack of consensus, or improved due to the need to face common challenges. All these perspectives are different sides of the same reality, marked by complexity and lack of knowledge on the part of trainee teachers. Owing to the emergency to address shared fronts, there has been an unprecedented exercise in global cooperation, but at the same time a sense of helplessness and lack of autonomy in schools has emerged. As we said, different capacities and experience between schools may be behind this disparity between perceptions.

In relation to the impact of COVID-19 on the community, respondents noted an almost total decrease in extracurricular activity during the quarantine, which gradually returned to normal activity (within the new normality) over the following months. While attendance at school and after-school activities does not have a significant impact on pupils’ morale [23], respondents noted the risks involved, in particular, the lack of reinforcement of school content that serves pupils who lag behind. Likewise, they also identified an increase in attitudes of solidarity, highlighting the close collaboration established between different educational agents with town halls, collectives, the police, the administration, and other associated agents.

Finally, with regard to their own perception of their abilities to deal with the educational reality, 60% of undergraduate students considered that they did not receive sufficient training to deal with the impact of COVID-19 on relations between educational agents, compared to 30% who said they felt prepared. Despite this, the vast majority considered their work placement in a pandemic context as an opportunity to learn more about internal school relations. They stated that it helped them to feel more self-confident, and they consider themselves more mature and prepared. This contrasts with the initial feelings of insecurity and uncertainty that arise at the moment of being introduced to a school reality different from the one shown during the degree. The internship period helps to reduce the more negative feelings of the new trainee teachers [30], although they all agree in pointing out the main weaknesses and shortcomings in their training. They all call for more training in health education, specifically on pediatric diseases, their prevention and treatment, and on specific cases of special education, which during the pandemic has shown the need for face-to-face teaching, due to the need to establish specific pedagogical links, because students are not the same in front of screens as they are when they sit at their desks [54]. They also call for more training to improve relations between school staff, as well as with the administration. Finally, many pointed out the need to expand their pedagogical training, especially to deal with exceptional scenarios, such as the pandemic.

## 5. Conclusions

The pandemic caused by COVID-19 left its mark on all levels of society and individuals. Additionally, in this case, the impact on the normal functioning of schools is no less evident, closed at first, then reopened with restrictions, and subject to instability. The trainee teachers were introduced for the first time in a school with no previous experience other than their time in an Infant School during their first years of life. This means almost no experience at all, marked by the memories that other people may have instilled in them, as well as by the emotions that arise from blurred and inscrutable pasts.

In general terms, trainee teachers have identified the main consequences of the pandemic as identified by Van Prooijen, Spadaro and Wang: reduced trust between strangers, reduced cooperation and prosocial behavior, and increased prejudice, conflict and polarization [16]. It is about collective effects resulting from the impact of the pandemic on individuals’ emotional health and cognitive functioning, leading to changes in behavior and social behaviors [17]. Its consequences on the school, and on the personal relationships of its staff, were identified by the majority of respondents.

It is worth highlighting the lack of knowledge that trainee teachers have about the role of the different educational administrations, as well as their competencies and communication channels. This poses a challenge for the university training they receive, which must consider the role of the teacher as the most important link between pupils, families and the rest of the agents and administrations. A better training that helps them to improve the relationship in the educational community also contributes to improving students’ academic performance. In this sense, this need arises as a pedagogical urgency, not because of the implications derived from healthy and fluid relations between agents, but as a *sine qua non* condition to guarantee the maximum benefit of educational communities. A condition that, as we have said, results in better academic performance of students, but also facilitates and improves teaching practice.

It is also noteworthy that respondents expressed their own teaching limitations. Specifically, they point to a lack of preparation for dealing with extraordinary teaching scenarios, such as the one posed by the pandemic. Additionally, noteworthy is the expressed need to promote ongoing training to help teachers not only to cope in virtual environments, but also to maintain the pedagogical objectives beyond the scenario in which the educational process is carried out. In short, the needs and demands presented pose a challenge for university education, which must reconfigure its academic offerings to integrate the new knowledge and skills that enable teachers to address the challenges associated with their labor.

## Data Availability

The data are not publicly available because of privacy or ethical restrictions.
